# NLRP3 deletion inhibits inflammation-driven mouse lung tumorigenesis induced by benzo(a)pyrene and lipopolysaccharide

**DOI:** 10.1186/s12931-019-0983-4

**Published:** 2019-01-29

**Authors:** Li Huang, Shuyin Duan, Hua Shao, Aihua Zhang, Shuang Chen, Peng Zhang, Na Wang, Wei Wang, Yongjun Wu, Jing Wang, Hong Liu, Wu Yao, Qiao Zhang, Feifei Feng

**Affiliations:** 1 Department of Toxicology, College of Public Health, Zhengzhou University, No.100 Kexue Avenue, Zhengzhou, 450001 Henan province China; 2 Department of Bone and Soft Tissue Cancer, Cancer, the Affiliated Cancer Hospital of Zhengzhou University (Henan Cancer Hospital), Zhengzhou, Henan China; 3 Department of Nephrology, the Children’s Hospital of Nanjing Medical University, Nanjing, Jiangsu China; 4 Institute of Pediatrics, Nanjing Medical University, Nanjing, Jiangsu China; 5 Department of Pulmonary Medicine, the First Affiliated Hospital of Zhengzhou University, Zhengzhou, Henan China

**Keywords:** Benzo(a)pyrene, Lipopolysaccharide, Lung tumorigenesis, Inflammation, NLRP3

## Abstract

**Background:**

Inflammatory micro-environment has been proposed to play a critical role in lung tumorigenesis. NLRP3 is known as an intracellular receptor involving inflammation and has been reported which is increasingly associated with tumor development, but the role in inflammation-driven lung cancer has not been fully clarified. In this study, we investigated whether lipopolysaccharide (LPS)-induced pulmonary inflammation could contribute to lung tumorigenesis induced by benzo(a)pyrene [B(a)p] in C57BL/6J mice and the role of NLRP3 in the pathogenesis.

**Methods:**

NLRP3−/− mice and C57BL/6J mice (wide-type, WT) were instilled intratracheally with B(a)p (1 mg/mouse) once a week for 4 times [the week of the last time of B(a)p treatment named Week 0], and mice were then instilled intratracheally with LPS at Week 3, 2.5 μg/mouse, once every three weeks for 5 times. At Week 30, the incidence, number, size and histopathology of lung tumor were analyzed.

**Results:**

Mice exposed to B(a)p or B(a)p plus LPS could induce lung tumors, whereas LPS or vehicles treatment could not induce lung tumorigenesis. In WT mice, B(a)p plus LPS exposure significantly increased tumor incidence, mean tumor count and tumor size of visible tumors of lungs compared with B(a)p treatment alone, and NLRP3 deletion inhibited lung tumorigenesis induced by B(a)p or B(a)p plus LPS. Histopathological examination found LPS-induced pulmonary inflammatory changes enhanced lung tumorigenesis induced by B(a)p in WT mice, deletion of NLRP3 improved the inflammatory changes induced by LPS and the number and size of pathological tumor nests induced by B(a)p or B(a)p plus LPS. In addition, we found B(a)p treatment and B(a)p plus LPS treatment predominately induced the development of adenoma.

**Conclusion:**

LPS enhanced B(a)p-induced lung tumorigenesis in WT and NLRP3−/− mice of C57BL/6J strain, and NLRP3 deletion inhibits lung tumorigenesis induced by B(a)p or B(a)p plus LPS.

## Introduction

Lung cancer is the leading cause of cancer-related mortality, which accounts for one-quarter of all cancer deaths [1]. Growing evidence demonstrated that tobacco smoke and air pollution are all main causes of lung cancer [2, 3]. Moreover, the promoting role of inflammation in lung cancer has been reported [4, 5]. Indeed, chronic obstructive pulmonary disease (COPD), pulmonary inflammatory diseases, was associated with higher lung cancer risk [6]. A chronic inflammatory micro-environment in the lung has been proposed to play a critical role in lung tumorigenesis. Lipopolysaccharide (LPS), a major proinflammatory component, can induce pulmonary inflammatory response, such as inflammatory cells infiltration, inflammatory cytokine release and so on, by activating NF-κB and NLRP3 signaling pathways [7, 8]. An earlier study has shown that LPS-induced pulmonary inflammatory changes in mouse enhance lung tumorigenesis induced by 4-(methylnitrosamino)-1-(3-pyridyl)-1-butanone (NNK) [9]. Benzo(a)pyrene [B(a)p] is the identified and important complete lung carcinogens and the carcinogenic capacity has been reported in studies both in vitro and in vivo [10, 11]. Although LPS-induced pulmonary inflammation could be a critical contributor to the induction of genotoxicity by B(a)p [12], the molecular mechanisms responsible for the enhancement of pulmonary inflammation in lung tumorigenesis have not been fully clarified.

NLRP3, a subset of the NOD-like receptor (NLR) family, can assemble with the adaptor apoptosis-associated speck-like protein (ASC) and procaspase-1 to form the NLRP3 inflammasome, which results in caspase-1 activation, and then leads to the release of interleukin (IL)-1β and IL-18 and the induction of pyroptosis [13]. For NLRP3 inflammasome, it has been reported to be involved in the pathologic process of inflammatory diseases, such as chronic airway diseases, inflammatory bowel diseases and so on [14, 15]. Furthermore, it was well documented that NLRP3 inflammasome plays a vital role in tumor development [16]. Multiple studies have demonstrated that NLRP3 inflammasome is largely protective in an inflammatory model of colorectal cancer [17, 18]. In addition, the promotive role of NLRP3 inflammasome in lung metastases, breast cancer and gastric carcinoma also has been reported [19]. However, the role of NLRP3 inflammasome in lung tumorigenesis, especially in inflammation-related lung tumorigenesis, remains unclear.

In this study, we investigated whether NLRP3 inflammasome play a detrimental role in promoting lung tumorigenesis using NLRP3−/− mice and C57BL/6J mice. To model inflammation-driven lung cancer, mice were instilled with B(a)p and then treated with LPS. Our findings could provide a novel therapeutic strategy for the treatment of lung cancer.

## Materials and methods

### Animals

NLRP3−/− mice on a C57BL/6J background were obtained as gift generously from Professor Aihua Zhang in Nanjing Children’s Hospital to explore the role of NLRP3 in the mouse model of inflammation-driven lung cancer. C57BL/6J mice (wide-type, WT) were bred from their heterozygous littermates in the College of Public Health of Zhengzhou University, Henan, China, and raised in stainless steel cages under standard conditions and allowed food and water ad libitum. The temperature was maintained at 22 °C, and the lights began from 08:00 to 20:00. All experimental procedures were approved by the Life Science Institutional Review Board of Zhengzhou University and performed strictly in accordance with the Guideline of Zhengzhou University for Animal Experiments.

### Inflammation-driven lung cancer mouse model

**Fig. 1:**
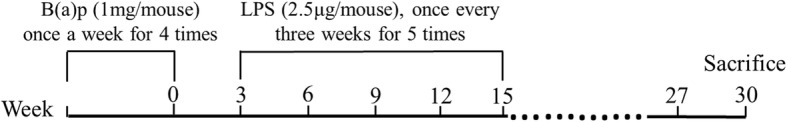
Experimental design of the study. NLRP3−/− mice and WT mice were randomly divided into four treatment groups, respectively. As follows: Group 1 (*n* = 15): Vehicle control; Group 2 (*n* = 15): LPS; Group 3 (*n* = 35): B(a)p; Group 4 (*n* = 35): B(a)p plus LPS. NLRP3−/− mice and WT mice, 6–8 weeks old, were instilled intratracheally with B(a)p once a week for 4 times and instilled intratracheally with LPS once every three weeks for 5 times since Week 3. Thirty weeks after the week of the last dose of B(a)p, mice were anaesthesia by pentobarbital sodium

NLRP3−/− mice and WT mice (half male and female, approximately 6–8 weeks old, 20-30 g) were randomly divided into four treatment groups, respectively, and treated as shown in Fig. 1. In detail, mice in groups 3 (*n* = 35) and 4 (*n* = 35) were instilled intratracheally with B(a)p (1 mg/mouse, dissolved in 50 μl glyceryl trioctanoate) once a week for 4 times [the week of the last time of B(a)p treatment named Week 0], whereas mice in group 1 (*n* = 15) were administered 50 μl glyceryl trioctanoate in a similar manner. At Week 3, mice in groups 2 (*n* = 15) and 4 were instilled intratracheally with LPS at a dose of 2.5 μg/mouse in 50 μL saline once every three weeks for 5 times. All instillations were performed under anesthesia with isoflurane (Sigma). The mice were anaesthesia by pentobarbital sodium (100 mg/kg) at Week 30. The lungs were harvested, and visible tumors on the surface of the lung were counted and the size of lung tumors was accessed using a straightedge. The left lobes of the lungs of group 1–4 were fixed in 4% paraformaldehyde for histopathological studies and the right lobes were stored at − 80 °C for subsequent assays. To assess the effect of the vehicle (glyceryl trioctanoate), NLRP3−/− mice (*n* = 6) and WT mice (*n* = 6) without any treatment (half male and female, approximately 6–8 weeks old, 20-30 g) were as normal control, the left lobes of the lungs of normal control mice were also collected and fixed for histopathology when they were fed for 34 weeks.

### Lung coefficient

The mice were euthanized at Week 30 and the lungs were removed from sacrificed mice. Whole lung tissues were washed in saline solution, sucked dry with filter paper, and then weighted. Lung coefficient was expressed as the percentage of the weight of respective lung of that mouse in the body weight of that mouse.

### Examination of lung pathological alterations

The left lobes of the lungs were placed in 4% paraformaldehyde overnight. After fixation, the lung tissues were embedded in paraffin, and cut into the sections of 5 μm in thickness to stain with haematoxylin and eosin (HE).

Lung tumors were classified as adenoma or squamous cell carcinoma based on those published by Nikitin et al. [20], and all the subtypes of lung cancer of mice were assessed independently by two experienced pathologists in a blinded manner. Adenoma, well circumscribed areas consisting of cuboidal to columnar cells lining alveoli, was usually small in size and retained preexisting alveolar structure, and which included solid, papillary and mixed subtypes. However, the hallmarks of squamous cell carcinoma are the differentiation features of the squamous epithelium: keratinization and intercellular bridges.

### Transmission electron microscopy

To evaluate the carcinogenesis capacity of B(a)p, the visible tumors on the surface of the lung were collected, fixed in 2.5% glutaraldehyde to overnight at 4 °C and postfixing in 1% O_S_O_4_–0.1 mol/l phosphate buffer. Then, ultrathin sections (60 nm) were cut on a microtome, placed on copper grids, stained with uranyl acetate and lead citrate, and exminrd in an electron microscope.

### Statistical analysis

Results were presented as mean ± SEM. Data comparison was carried out by two-tailed Student’s t-test and one-way ANOVAs using SPSS21.0 (IBM, NC, USA). A two-tailed *P* value < 0.05 was considered statistically significant.

## Results

### NLRP3 deletion inhibited lung tumorigenesis induced by B(a)p plus LPS in mice

**Fig. 2:**
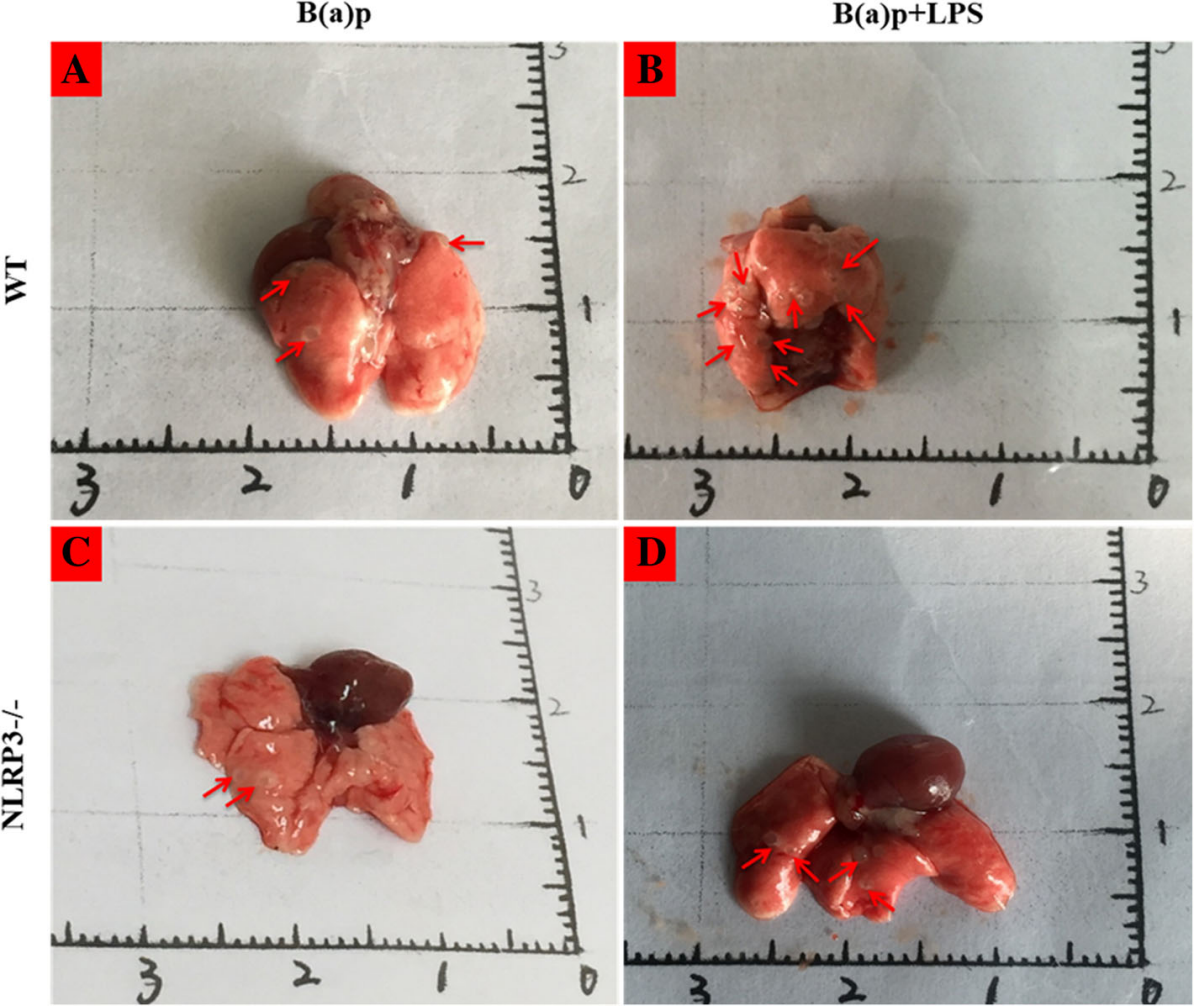
B(a)p and B(a)p plus LPS exposure induced lung tumors. Representative lung nodules seen in WT mice (**a**, **b**) and NLRP3−/− mice (**c**, **d**) induced by B(a)p or B(a)p plus LPS. Red arrows show visible tumors on the surface of the lung

**Fig. 3:**
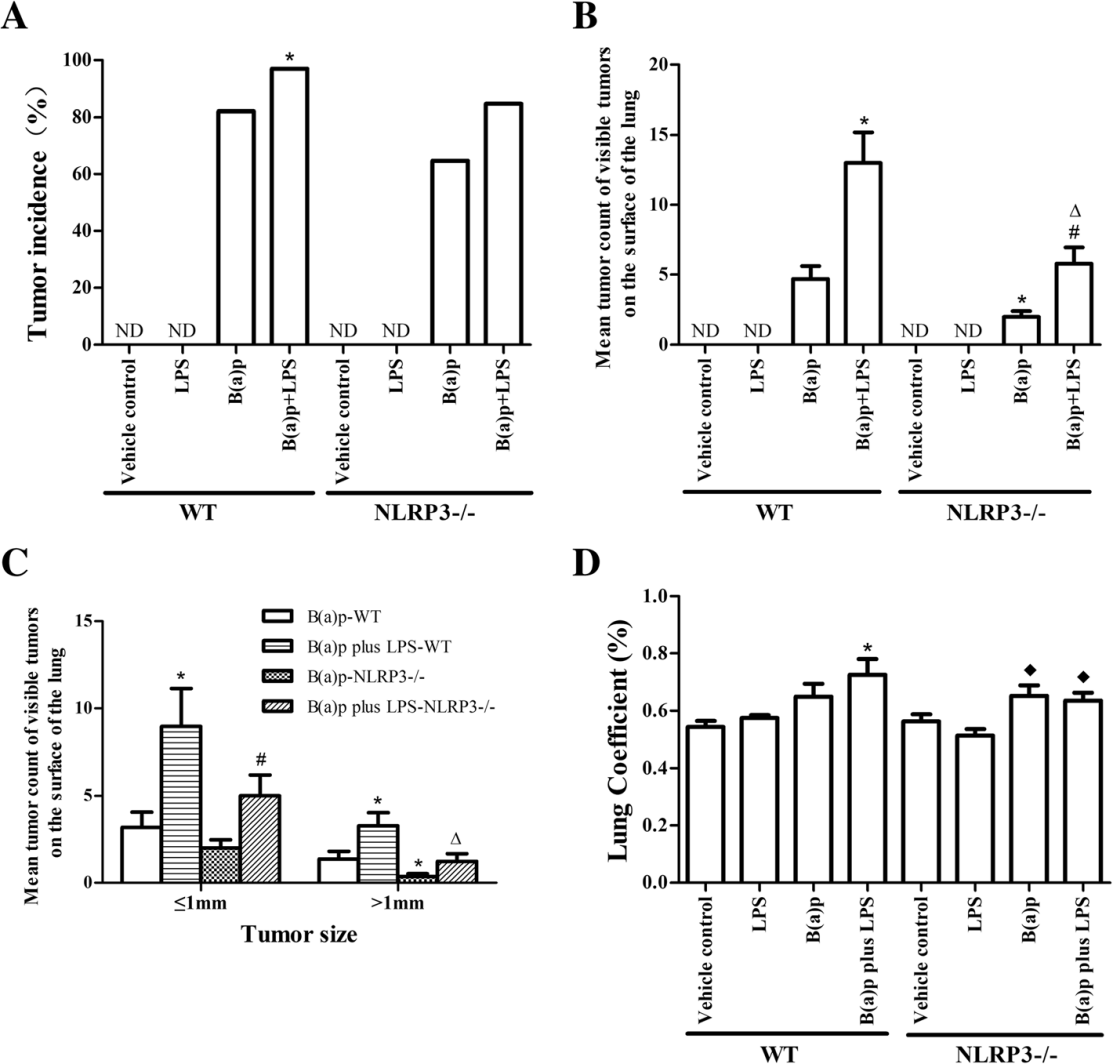
B(a)p and B(a)p plus LPS induced lung tumorigenesis in WT mice and NLRP3−/− mice**.** The tumor incidence (**a**), mean tumor count (**b**) and tumor size (**c**) of visible tumors on the surface of the lung from B(a)p-treated and B(a)p plus LPS in WT mice and NLRP3−/− mice. **d** Lung coefficient of WT mice and NLRP3−/− mice exposed to vehicle, LPS, B(a)p or B(a)p plus LPS. *: vs B(a)p-WT, *P* < 0.05;#: vs B(a)p-NLRP3−/−, *P* < 0.05; ∆: vs B(a)p plus LPS-WT, *P* < 0.05, ♦: vs LPS-WT, *P* < 0.05. ND: Not Detectable

As shown in Fig. 2 and Fig. 3, mice exposed to B(a)p or B(a)p plus LPS could induce lung tumors, whereas LPS or vehicles treatment could not induce lung tumorigenesis. In WT mice, the tumor incidence of mice exposed to B(a)p plus LPS (96.97%) was increased compared with mice exposed to B(a)p alone (82.05%)(*P* < 0.05) (Fig. 3a). Moreover, mice treated with B(a)p plus LPS developed 13.0 ± 12.4 visible tumors/mouse on the surface of the lung, which was significantly higher compared with mice treated with B(a)p alone (4.7 ± 5.7 tumors/mouse)(*P* < 0.05) (Fig. 3b). The size of visible tumors on the surface of the lung was assessed with two size categories: ≤1 mm and > 1 mm. As shown in Fig. 3c, smaller tumors (≤1 mm) were more abundant in the B(a)p plus LPS treatment than in B(a)p treatment alone (*P* < 0.05). Also, the frequency of larger tumors (> 1 mm) was significantly higher in mice exposed to B(a)p plus LPS than in mice exposed to B(a)p alone (*P* < 0.05). These results indicate LPS enhances B(a)p-induced lung tumorigenesis, suggesting that this study successfully developed a mouse model of inflammation-driven lung tumorigenesis.

To determine if NLRP3 inflammasome plays a vital role in inflammation-driven lung tumorigenesis, we compared the tumor incidence, multiplicity and the size of visible tumors on the surface of the lung in WT mice and NLRP3−/− mice. Similarly, B(a)p plus LPS exposure significantly increased the tumor incidence, mean tumor count and the size of lung tumors than B(a)p exposure in NLRP3−/− mice. Importantly, the lung tumor multiplicity of NLRP3−/− mice exposed to B(a)p or B(a)p plus LPS was significantly less than WT mice treated with B(a)p or B(a)p plus LPS, respectively (*P* < 0.05). In addition, NLRP3 deletion mainly reduced the growth of B(a)p or B(a)p plus LPS-induced lung tumors (*P* < 0.05). Taken together, these results demonstrate that NLRP3 deletion significantly inhibits lung tumorigenesis induced by B(a)p or B(a)p plus LPS in mice.

### Effects of B(a)p plus LPS exposure on lung coefficient

Lung coefficient is also an indicator of lung injury in mice. As shown in Fig. 3d, b (a)p plus LPS treatment of WT mice induced the rise of lung coefficient compared with that in WT mice treated with vehicles (*P* < 0.05). In NLRP3−/− mice, lung coefficient in mice exposed to B(a)p and B(a)p plus LPS was significantly increased than mice exposed to LPS alone (*P* < 0.05), but there were no significant difference between WT mice and NLRP3−/− mice with B(a)p or B(a)p plus LPS treatment, respectively.

### Pathological alterations in the lungs of mice exposed to B(a)p plus LPS

**Fig. 4:**
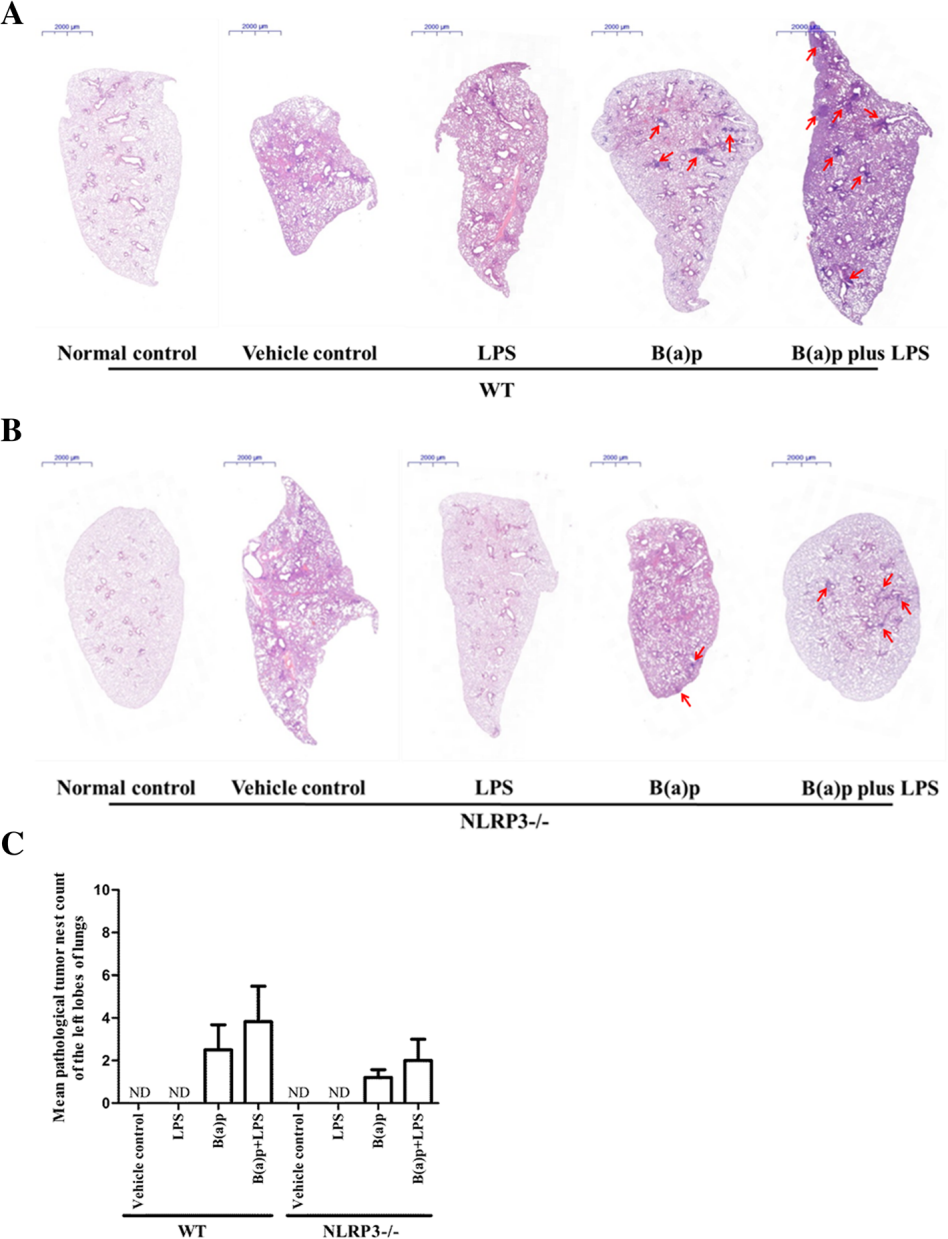
Pathological alterations in the cross-section of the left lobes of lungs of WT mice and NLRP3−/− mice. The pulmonary morphological alterations in the left lobes of lungs of WT mice (**a**) and NLRP3−/− mice (**b**) exposed to normal control, vehicle control, LPS, B(a)p or B(a)p plus LPS were evaluated by HE staining. **c** The number of pathological tumor nests in cross-section of the left lobes of lungs induced by B(a)p or B(a)p plus LPS in WT mice and NLRP3−/− mice. Red arrows show pathological tumor nests in cross-section of the left lobes of lungs. ND: Not Detectable

**Fig. 5:**
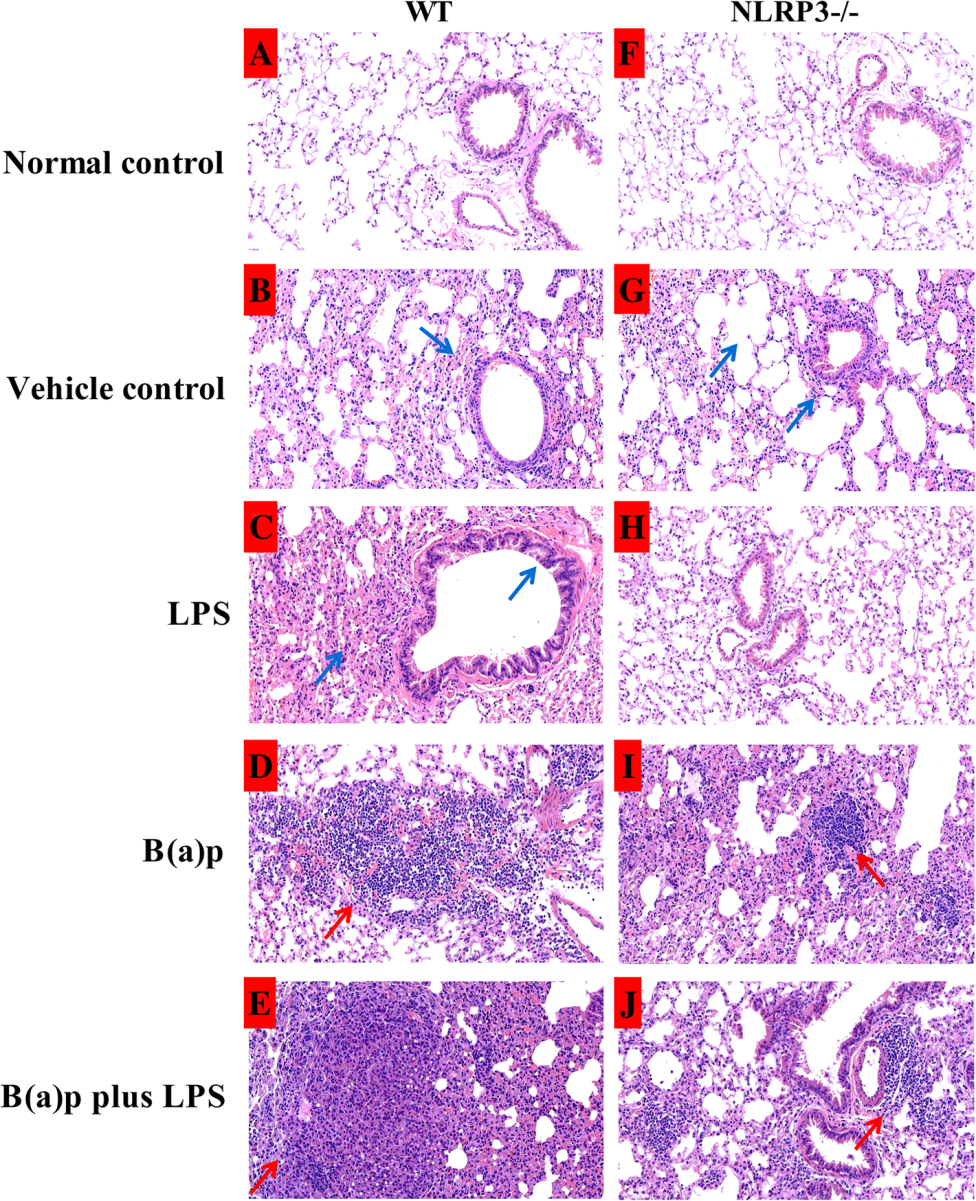
Pathological alterations in WT mice and NLRP3−/− mice with amplification (200×). Pulmonary pathological changes of WT mice (**a**-**e**) and NLRP3−/− mice (**f**-**j**) exposed to normal control, vehicle control, LPS, B(a)p or B(a)p plus LPS were evaluated by HE staining. **a**-**e**: pathological changes of WT mice in different treatment with amplification (200×); (**f**-**j**): pathological changes of NLRP3−/− mice in different treatment with amplification (200×). Blue arrows show inflammatory changes in mice exposed to LPS or vehicles including fractures of alveolar walls, infiltration of inflammatory cells and injury of bronchial epithelium; Red arrows show pathological tumor nests in mice exposed to B(a)p or B(a)p plus LPS

**Fig. 6:**
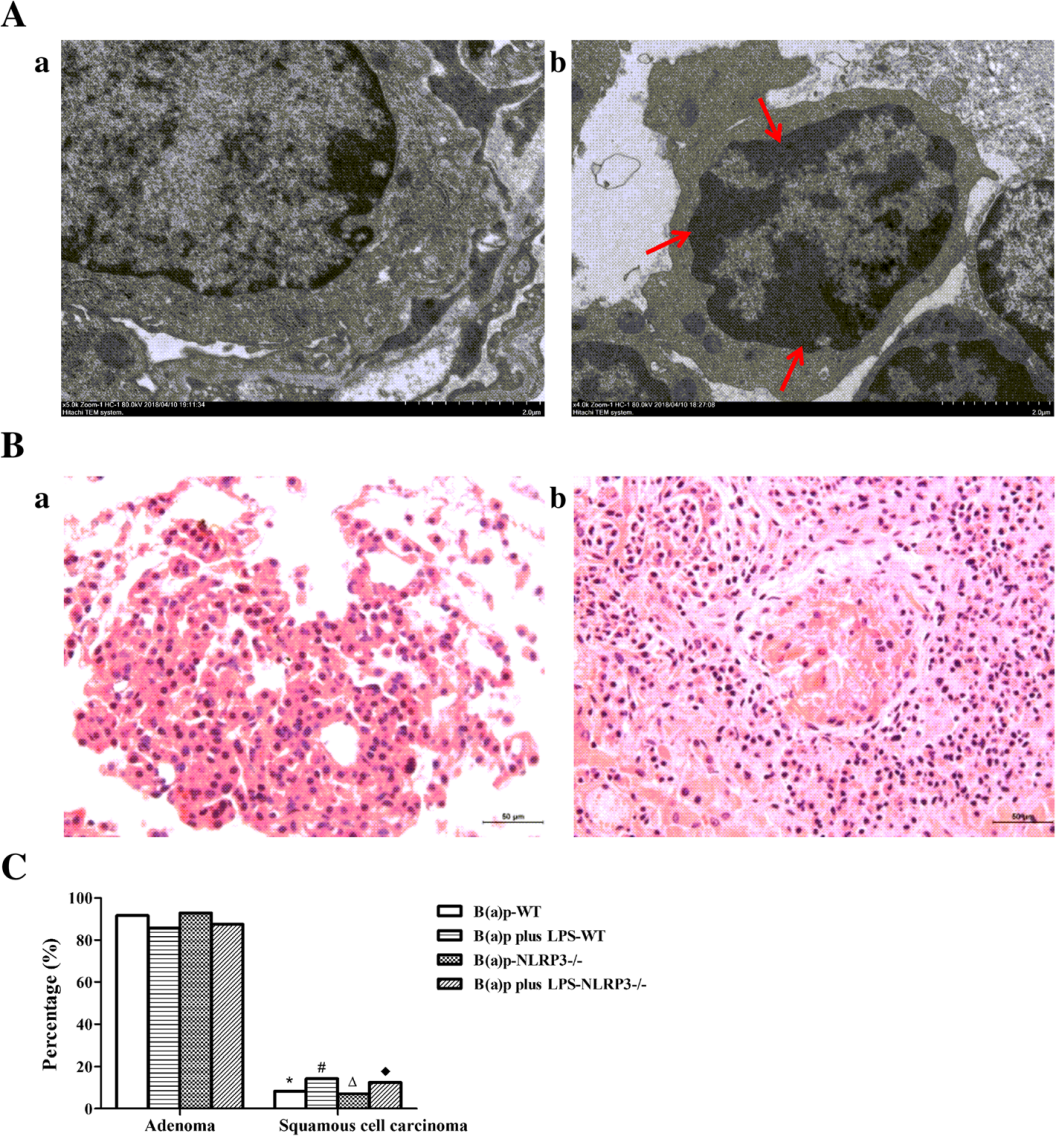
Lung adenoma and squamous cell carcinoma in WT mice and NLRP3−/− mice induced by B(a)p or B(a)p plus LPS. **A** Representative images of normal lung tissue (a) and tumor (b) by transmission electron microscopy in WT mice. **B** Representative images of lung adenoma (a) and squamous cell carcinoma (b) with amplification (200×). **C** The percentage of lung adenoma and squamous cell carcinoma induced by B(a)p or B(a)p plus LPS in WT mice and NLRP3−/− mice. Red arrows show heterochromatin in lung tumors induced by B(a)p plus LPS. *: vs B(a)p-WT, *P* < 0.05; #: vs B(a)p plus LPS-WT, *P* < 0.05; ∆: vs B(a)p-NLRP3−/−, *P* < 0.05, ♦: vs B(a)p plus LPS-NLRP3−/−, *P* < 0.05

As shown in Fig. 4 and Fig. 5, we found the significantly inflammatory changes in mice exposed to LPS or vehicles including fractures of alveolar walls, infiltration of inflammatory cells and injury of bronchial epithelium compared with the normal control group in WT mice. Interestingly, deletion of NLRP3 improved the inflammatory changes induced by LPS, but vehicles-induced inflammatory changes still persist. B(a)p or B(a)p plus LPS treatment induced the development of lung tumor. The number and size of pathological tumor nests in cross-section of the left lobes of lungs induced by LPS plus B(a)p in WT mice were increased than mice exposed to B(a)p alone, whereas deletion of NLRP3 attenuated the number and size of pathological tumor nests induced by B(a)p or B(a)p plus LPS compared with WT mice, respectively. In addition, as shown in Fig. 6a, we found WT mice exposed to B(a)p plus LPS induced lung tumorigenesis, which was evidenced by the formation of heterochromatin using transmission electron microscopy. These results indicated that LPS exposure induces marked lung inflammation and damage, and play a critical role in promoting B(a)p-induced lung tumorigenesis. Moreover, NLRP3 deletion prevented lung tumorigenesis induced by B(a)p or B(a)p plus LPS.

To determine the pathological type of lung tumors induced by B(a)p or B(a)p plus LPS, lung tissue sections were evaluated with HE staining. As depicted in Fig. 6B, B (a)p or B(a)p plus LPS exposure induced the development of lung adenoma and squamous cell carcinoma. In WT mice, the adenoma incidence of mice exposed to B(a)p (91.7%) or B(a)p plus LPS (85.7%) were significantly increased compared with the development of lung squamous cell carcinoma in respective treatment. Moreover, NLRP3−/− mice treated with B(a)p or B(a)p plus LPS also mainly developed lung adenoma with 92.9% and 87.5%, respectively (Fig. 6C). However, there was no significant difference in the adenoma incidence or the squamous cell carcinoma incidence between B(a)p treatment and B(a)p plus LPS treatment in WT mice and NLRP3−/− mice, respectively.

## Discussion

Chronic pulmonary inflammation can promote lung tumorigenesis, but the molecular pathways involved remain poorly defined. To better understand the association between pulmonary inflammation and lung cancer, inflammation-driven lung cancer mouse model was developed. LPS, a major proinflammatory glycolipid component of the gram-negative bacterial cell wall, is ubiquitously present in the environment. Studies have showed that LPS could significantly up-regulate the production of inflammatory cytokines via TLR4, NF-κB and NLRP3 signaling pathways [8, 21], and long-time LPS instillation in mice is reported to be associated with chronic lung inflammation and persistent pathologic alterations [22]. In addition, Tamene Melkamu et al. have established a mouse model of inflammation-driven lung cancer induced by LPS plus NNK using A/J mice, and found critical roles of PI3K/Akt, NF-kB and STAT3 signaling pathways involved in lung tumorigenesis [9]. Similarly, a mouse model of inflammation-driven lung squamous cell carcinoma (LSCC) with A/J mice was induced by Nnitroso-trischloroethylurea (NTCU) and enhanced by LPS [23]. A/J mice strain has been reported that it is sensitive to lung cancer induction by carcinogen and has a high incidence of spontaneous lung adenomas [24]. However, the C57BL/6J mice strain, a common inbred strain of laboratory mouse and the most widely used mouse strain for use as models of human disease, has a low susceptibility to tumors. To develop a facile mouse model of lung tumorigenesis in C57BL/6J mice strain, in this study, mice was instilled with B(a)p and LPS to induce lung tumorigenesis, and we found pulmonary inflammation induced by LPS enhanced B(a)p-induced lung tumorigenesis, as evidenced by increased tumor incidence, mean tumor count and tumor size of visible tumors on the surface of lungs, which was in line with the above reports. In addition, B(a)p and LPS exposure significantly increased lung coefficient compared with mice treated vehicles, and the increase in lung coefficient may be attributed to the developed tumor nodules. All these findings indicated that this study successfully developed a C57BL/6J mouse model of inflammation-driven lung tumorigenesis, which could provide a better study strategy to better understanding the effect of pulmonary inflammation on lung cancer through developing a knockout mouse model on a C57BL/6J background.

NLRP3 deletion significantly suppressed the tumor incidence, mean tumor count and tumor size of visible tumors on the surface of lungs induced by B(a)p or B(a)p plus LPS, implying NLRP3 inflammasome may be involved in the enhancement of pulmonary inflammation in lung tumorigenesis. It has been well documented that NLRP3 inflammasome was increasingly associated with tumor development, but its role in different cancer was inconsistent. Findings from previous studies demonstrated the protective role of NLRP3 inflammasome in colorectal tumorigenesis. Indeed, NLRP3 inflammasome-deficient mice, including NLRP3−/−, ASC−/− and Caspase-1−/− mice, were increased highly susceptible to colitis-associated colorectal tumor formation [25]. However, dextran sodium sulfate (DSS)-induced colitis could be mediated by the NLRP3 inflammasome activation-induced caspase-1 cleavage and IL-1β secretion, and mice lacking NLRP3 were significantly protected from colitis [26]. Another study using human lung adenocarcinoma A549 cells demonstrated that NLRP3 inflammasome activation can enhance the proliferation and migration of A549 cells [27]. In addition, NLRP3 inflammasome pathway contributed to the development and progression of last stage human melanoma cells [28], its promoted role in cancer formation was consistent with the present results.

Histopathological observation not only confirmed that LPS-induced lung inflammatory changes and pathological injury enhanced B(a)p-induced lung tumorigenesis, and NLRP3 deletion significantly improved these changes, but also found that B(a)p exposure and B(a)p plus LPS exposure predominately induced the occurrence of lung adenoma in this study. Indeed, it has been demonstrated that lung tumors induced by chemical carcinogens almost are pulmonary adenoma and adenocarcinomas, including polycyclic aromatic hydrocarbons, nitrosamines and so on [29]. B(a)p, the derivative of polycyclic aromatic hydrocarbons, can induce DNA damage through the enhanced formation of covalent B(a)p-DNA adducts, results in lung tumorigenesis [12]. In addition, the formation of heterochromatin also suggested the carcinogenic capacity of B(a)p by transmission electron microscopy. Thus, our results verified that B(a)p or B(a)p plus LPS could induce lung cancer, especially lung adenoma, and successfully provided some evidence regarding the role of NLRP3 in inflammation-driven lung tumorigenesis through our mouse models.

## Conclusions

Our results from this study demonstrated that LPS enhanced B(a)p-induced lung tumorigenesis in WT and NLRP3−/− mice of C57BL/6J strain, and NLRP3 deletion inhibits lung tumorigenesis induced by B(a)p or B(a)p plus LPS. However, the mechanism of NLRP3 expression and NLRP3 inflammasome activation in inflammation-related lung cancer should further investigate.

## Abbreviations

ASC: Apoptosis-associated speck-like protein
B(a)p: Benzo(a)pyrene
COPD: Chronic obstructive pulmonary disease
DSS: Dextran sodium sulfate
IL: Interleukin
LPS: Lipopolysaccharide
LSCC: Lung squamous cell carcinoma
NLR: NOD-like receptor
NNK: 4-(methylnitrosamino)-1-(3-pyridyl)-1-butanone
NTCU: Nnitroso-trischloroethylurea
WT: Wide-type

## References

[1] Siegel RL, Miller KD, Jemal A (2018). Cancer statistics, 2018. CA Cancer J Clin.

[2] Hong QY, Wu GM, Qian GS, Hu CP, Zhou JY, Chen LA, Li WM, Li SY, Wang K, Wang Q (2015). Prevention and management of lung cancer in China. Cancer.

[3] Warren GW, Cummings KM. Tobacco and lung cancer: risks, trends, and outcomes in patients with cancer. Am Soc Clin Oncol Educ Book. 2013:359–64. 10.14694/EdBook_AM.2013.33.359 23714547

[4] Hystad P, Demers PA, Johnson KC, Carpiano RM, Brauer M (2013). Long-term residential exposure to air pollution and lung cancer risk. Epidemiology.

[5] Shi L, Wang L, Hou J, Zhu B, Min Z, Zhang M, Song D, Cheng Y, Wang X (2015). Targeting roles of inflammatory microenvironment in lung cancer and metastasis. Cancer Metastasis Rev.

[6] Bozinovski S, Vlahos R, Anthony D, McQualter J, Anderson G, Irving L, Steinfort D (2016). COPD and squamous cell lung cancer: aberrant inflammation and immunity is the common link. Br J Pharmacol.

[7] Tian L, Li W, Wang T (2017). Therapeutic effects of silibinin on LPS-induced acute lung injury by inhibiting NLRP3 and NF-kappaB signaling pathways. Microb Pathog.

[8] Zhang A, Wang S, Zhang J, Wu H (2016). Genipin alleviates LPS-induced acute lung injury by inhibiting NF-kappaB and NLRP3 signaling pathways. Int Immunopharmacol.

[9] Melkamu T, Qian X, Upadhyaya P, O'Sullivan MG, Kassie F (2013). Lipopolysaccharide enhances mouse lung tumorigenesis: a model for inflammation-driven lung cancer. Vet Pathol.

[10] Huang H, Pan X, Jin H, Li Y, Zhang L, Yang C, Liu P, Liu Y, Chen L, Li J (2015). PHLPP2 downregulation contributes to lung carcinogenesis following B[a]P/B[a]PDE exposure. Clin Cancer Res.

[11] Kasala ER, Bodduluru LN, Barua CC, Sriram CS, Gogoi R (2015). Benzo(a)pyrene induced lung cancer: role of dietary phytochemicals in chemoprevention. Pharmacol Rep.

[12] Arlt VM, Krais AM, Godschalk RW, Riffo-Vasquez Y, Mrizova I, Roufosse CA, Corbin C, Shi Q, Frei E, Stiborova M (2015). Pulmonary inflammation impacts on CYP1A1-mediated respiratory tract DNA damage induced by the carcinogenic air pollutant benzo[a]pyrene. Toxicol Sci.

[13] He Y, Hara H, Nunez G (2016). Mechanism and regulation of NLRP3 Inflammasome activation. Trends Biochem Sci.

[14] Im H, Ammit AJ (2014). The NLRP3 inflammasome: role in airway inflammation. Clin Exp Allergy.

[15] Kim JM (2011). Inflammatory bowel diseases and inflammasome. Korean J Gastroenterol.

[16] Kantono M, Guo B (2017). Inflammasomes and Cancer: the dynamic role of the Inflammasome in tumor development. Front Immunol.

[17] Allen IC, TeKippe EM, Woodford RM, Uronis JM, Holl EK, Rogers AB, Herfarth HH, Jobin C, Ting JP (2010). The NLRP3 inflammasome functions as a negative regulator of tumorigenesis during colitis-associated cancer. J Exp Med.

[18] Dupaul-Chicoine J, Yeretssian G, Doiron K, Bergstrom KS, McIntire CR, LeBlanc PM, Meunier C, Turbide C, Gros P, Beauchemin N (2010). Control of intestinal homeostasis, colitis, and colitis-associated colorectal cancer by the inflammatory caspases. Immunity.

[19] Karki R, Man SM, Kanneganti TD (2017). Inflammasomes and Cancer. Cancer Immunol Res.

[20] Nikitin AY, Alcaraz A, Anver MR, Bronson RT, Cardiff RD, Dixon D, Fraire AE, Gabrielson EW, Gunning WT, Haines DC (2004). Classification of proliferative pulmonary lesions of the mouse: recommendations of the mouse models of human cancers consortium. Cancer Res.

[21] Xiang P, Chen T, Mou Y, Wu H, Xie P, Lu G, Gong X, Hu Q, Zhang Y, Ji H (2015). NZ suppresses TLR4/NF-kappaB signalings and NLRP3 inflammasome activation in LPS-induced RAW264.7 macrophages. Inflamm Res.

[22] Vernooy JH, Dentener MA, van Suylen RJ, Buurman WA, Wouters EF (2002). Long-term intratracheal lipopolysaccharide exposure in mice results in chronic lung inflammation and persistent pathology. Am J Respir Cell Mol Biol.

[23] Song JM, Qian X, Teferi F, Pan J, Wang Y, Kassie F (2015). Dietary diindolylmethane suppresses inflammation-driven lung squamous cell carcinoma in mice. Cancer Prev Res (Phila).

[24] Beer DG, Malkinson AM (1985). Genetic influence on type 2 or Clara cell origin of pulmonary adenomas in urethan-treated mice. J Natl Cancer Inst.

[25] Zaki MH, Vogel P, Body-Malapel M, Lamkanfi M, Kanneganti TD (2010). IL-18 production downstream of the Nlrp3 inflammasome confers protection against colorectal tumor formation. J Immunol.

[26] Bauer C, Duewell P, Mayer C, Lehr HA, Fitzgerald KA, Dauer M, Tschopp J, Endres S, Latz E, Schnurr M (2010). Colitis induced in mice with dextran sulfate sodium (DSS) is mediated by the NLRP3 inflammasome. Gut.

[27] Wang Y, Kong H, Zeng X, Liu W, Wang Z, Yan X, Wang H, Xie W (2016). Activation of NLRP3 inflammasome enhances the proliferation and migration of A549 lung cancer cells. Oncol Rep.

[28] Okamoto M, Liu W, Luo Y, Tanaka A, Cai X, Norris DA, Dinarello CA, Fujita M (2010). Constitutively active inflammasome in human melanoma cells mediating autoinflammation via caspase-1 processing and secretion of interleukin-1beta. J Biol Chem.

[29] Meuwissen R, Berns A (2005). Mouse models for human lung cancer. Genes Dev.

